# A description of a knowledge broker role implemented as part of a randomized controlled trial evaluating three knowledge translation strategies

**DOI:** 10.1186/1748-5908-4-23

**Published:** 2009-04-27

**Authors:** Maureen Dobbins, Paula Robeson, Donna Ciliska, Steve Hanna, Roy Cameron, Linda O'Mara, Kara DeCorby, Shawna Mercer

**Affiliations:** 1 School of Nursing, McMaster University, Hamilton, Canada; 2 Department of Clinical Epidemiology and Biostatistics and CANCHILD Centre, McMaster University, Hamilton, Canada; 3 Lyle Hallman Institute, University of Waterloo, Waterloo, Canada; 4 The Guide to Community Preventive Services, National Center for Health Marketing, Centers for Disease Control and Prevention, Atlanta, USA

## Abstract

**Background:**

A knowledge broker (KB) is a popular knowledge translation and exchange (KTE) strategy emerging in Canada to promote interaction between researchers and end users, as well as to develop capacity for evidence-informed decision making. A KB provides a link between research producers and end users by developing a mutual understanding of goals and cultures, collaborates with end users to identify issues and problems for which solutions are required, and facilitates the identification, access, assessment, interpretation, and translation of research evidence into local policy and practice. Knowledge-brokering can be carried out by individuals, groups and/or organizations, as well as entire countries. In each case, the KB is linked with a group of end users and focuses on promoting the integration of the best available evidence into policy and practice-related decisions.

**Methods:**

A KB intervention comprised one of three KTE interventions evaluated in a randomized controlled trial.

**Results:**

KB activities were classified into the following categories: initial and ongoing needs assessments; scanning the horizon; knowledge management; KTE; network development, maintenance, and facilitation; facilitation of individual capacity development in evidence informed decision making; and g) facilitation of and support for organizational change.

**Conclusion:**

As the KB role developed during this study, central themes that emerged as particularly important included relationship development, ongoing support, customized approaches, and opportunities for individual and organizational capacity development. The novelty of the KB role in public health provides a unique opportunity to assess the need for and reaction to the role and its associated activities. Future research should include studies to evaluate the effectiveness of KBs in different settings and among different health care professionals, and to explore the optimal preparation and training of KBs, as well as the identification of the personality characteristics most closely associated with KB effectiveness. Studies should also seek to better understand which combination of KB activities are associated with optimal evidence-informed decision making outcomes, and whether the combination changes in different settings and among different health care decision makers.

## Background

While there are some recent systematic reviews regarding strategies to change health care practitioner behaviour [1-3], there are currently no definitive answers of how best to move toward 'evidence-informed' public health decision making. It is believed however, that the incorporation of the best available evidence into health policy and practice decisions would result in optimal patient and population health outcomes [4]. Currently, the evidence demonstrates that traditional one-way passive strategies used alone are relatively ineffective [5,6]. Strategies that are more interactive and involve face-to-face contact show promising results [5,7-11], and involvement of decision makers in the research process is associated with a higher degree of research uptake [12,13]. One hypothesis emerging from the literature is that a combination of strategies, such as an interactive KTE approach that reinforces relationships between researchers and users, and reaches potential users on multiple levels interacting face-to-face, may be most effective in achieving evidence-informed decision making [14,15].

A KB is a popular emerging KTE strategy to promote interaction between researchers and end users, as well as to develop capacity for evidence-informed decision making (EIDM). Although the health care literature is sparse with evaluations of KB impact [16], there is considerable evidence in other fields, particularly the business and agricultural sectors [17-23].

A KB provides a link between research producers and end users by developing a mutual understanding of goals and cultures, collaborates with end users to identify issues and problems for which solutions are required [24], and facilitates the identification, access, assessment, interpretation, and translation of research evidence into local policy and practice [16,17,25-27]. KBs also facilitate knowledge exchange, build rapport with target audiences, forge new connections across domains [28-31], and assess end users, whether they be individuals or organizations, to identify their strengths, knowledge, and capacity for evidence-informed decision making [32], in order to better tailor KB interventions to their specific needs. Knowledge brokering can be carried out by individuals [16,20,27,33], groups and/or organizations [4,23,29], and entire countries [34]. In each case, the KB is linked with a group of end users and focuses on promoting the integration of the best available evidence into policy and practice-related decisions. A key attribute of the KBs is their skill in the interpretation and application of research.

The KB also synthesizes local community and patient data with general and specific research knowledge to assist users in translating the evidence into locally relevant recommendations for policy and practice. An important component related to the success of this activity is the KB's ability to tailor the key messages from research evidence to the local/regional perspective, while also ensuring the 'language' used is meaningful for different end users [4,8,29,35,36]. Another key component is the KB's ability to develop a trusting and positive relationship with end users and to assist them to incorporate research evidence in their policy and practice decisions [17,37-39], while at the same time promoting exchange of knowledge such that researchers and users become more appreciative of the context of each other's work.

In order to incorporate appropriate forms of knowledge at the appropriate times, KBs need to be attuned to their audience as well as their audience's environment. KBs then work to facilitate organizational change [24,31], eliminate environmental barriers to evidence-informed decision making (EIDM) [40], and promote an organizational culture that values the use of the best available evidence in policy and practice [17,25,41]. Political and infrastructure support for EIDM are seen as important precursors for the incorporation of research evidence into decision making [21,25], and hence the KB must focus on ensuring adequate support for EIDM to be achieved. Finally, creating networks of people with common interests is a key KB activity [17,20,32,41,42], and has been shown to be an integral [43,44] and effective [45] component of knowledge brokering.

The KB role is a unique and challenging one, and few people currently possess the skills necessary to be effective in this position. It is also unknown to what extent these skills and attributes can be taught. However, to be successful KBs require superior interpersonal skills [26,46,47] communication skills [16,31,32,41,47], and motivational skills [32], and should possess expertise from both end users' and researchers' domains [12,17,41,47,48]. Furthermore, a KB requires expertise in gathering evidence, critically appraising evidence, synthesizing information, and interpreting the information in terms of the bigger picture. In terms of personality attributes, a KB should be someone who is a skilled mediator and team builder while being flexible and diplomatic with excellent business and communication skills [16].

Anecdotal evidence suggests that knowledge brokering can be effective in improving the quality and use of evidence in healthcare decision making [25,41]. While the number of published papers discussing knowledge brokering has grown dramatically; few have studied the impact of KBs on EIDM using scientific approaches [26]. The purpose of this paper is to describe in detail the KB intervention that comprised one of three KTE interventions evaluated in a randomized controlled trial (RCT) and to reflect on the future development of the role in public health as well as other health care settings. While the overall finding from the RCT demonstrated that tailored messaging was more effective, under certain circumstances, compared to knowledge brokering or access to an online registry of synthesized evidence, there was evidence that knowledge brokering had a significant positive effective for public health departments that perceived their organization did not value the use of research evidence in decision making. The results of the RCT have been submitted for publication elsewhere (Dobbins M, Robeson P, Ciliska D, Hanna S, Manske S, Cameron R, Mercer S, O'Mara L, DeCorby K., A randomized controlled trial evaluating the impact of knowledge translation and exchange strategies, submitted).

## Methods

A stratified RCT was conducted among Canadian public health departments. Public health departments in Canada are responsible for promoting the health of the population, preventing disease, and providing medical care to treat communicable diseases. They provide services that focus on promoting prenatal, newborn, and parent health, as well as health promotion within schools and worksites, nutritional counselling, physical activity promotion, injury prevention, development of community strengths to promote and improve health, and the promotion of healthy environments [49]. All provinces and territories in Canada have recommendations in place requiring public health departments to develop and implement strategies to promote healthy body weight in children. Despite these recommendations there is limited capacity (*i.e*., time, skill, access) among public health decision makers and limited resources to utilize the best available research evidence with which to plan and implement effective healthy body weight programs and services.

The KTE interventions, implemented for one year in 2005, focused on promoting the uptake of effective public health strategies for promoting healthy body weight in children. One decision maker from each participating local or regional public health department was randomized to three intervention groups with progressively more active KTE strategies: access to an online registry of effectiveness evidence ; registry access and targeted messages; and registry access, targeted messages, and interaction with a KB. These decision maker participants were directly responsible for making decisions related to program planning or health policy for healthy body weight promotion in children in their public health department. In Ontario, relevant titles included program managers and/or coordinators, and in the rest of Canada program directors.

Following ethics approval and recruitment, organizations were stratified into three strata according to size of population served, and randomly allocated to one of the three groups using a computer generated random numbers table by a statistician external to the study. The primary unit of analysis was public health departments. The KB kept a daily journal in which all interactions were documented and reflections of the impact of these activities were noted. The journal provided the data used for describing the KB role in this paper. The primary investigator and KB reviewed the journal separately and came to consensus on the major themes identified in implementing the role.

## Results

### KB intervention

One KB working in a full time equivalent position provided knowledge brokering services to all English speaking participants allocated to the KB group (n = 30). A second Francophone KB (0.2 full time equivalent) provided KB services to French speaking participants allocated to the KB group n = 6). This paper reports the activities of the English speaking KB. Qualifications sought for the KB in this study included a Masters of Science (no particular field required), extensive knowledge of public health in Canada, some experience in research and in interpreting research results; experience in healthy body weight programming; and practical experience as a public health decision maker.

Specific tasks conducted by the KB included: ensuring relevant research evidence related to healthy body weight promotion was transferred to the public health decision makers in ways that were most useful to them, and assisting them in translating that evidence into local practice. This was accomplished primarily through electronic and telephone communication, along with a site visit of one to two days in length to each health department, and three day-long regional workshops. The KB maintained a daily reflective journal documenting all interaction with participants; reflecting on the interactions, what appeared to be working, and perceived impact of the KB activities. The data collected in the KBs journal allowed us to identify how much time was spent engaged in specific activities. Essentially, the total hours worked each week were tallied along with the total hours spent in the different KB roles. For example, twenty percent of KB time was spent facilitating knowledge and skill development either through face-to-face workshops or online strategies such as webinars, interactive web-enabled meetings, or conferences. Eighty percent of time was spent preparing for and directly interacting with participants. The proportion of time the KB spent preparing for interaction with participants was 40 to 50% early in the project, and declined to 30% as both public health decision makers and the KB became more skilled in their respective roles. KB activities were classified into the following categories, which will each be discussed in greater detail: initial and ongoing needs assessments; scanning the horizon; knowledge management; KTE; network development, maintenance, and facilitation; facilitation of individual capacity development in EIDM; and facilitation of and support for organizational change.

### Individual and organizational assessment

#### Baseline Assessment

At the start of the intervention, the KB conducted an assessment at the individual, organizational, and environmental levels, in order to identify strengths, knowledge, and capacity for EIDM. The development of the assessment tool was guided by Dobbins' Framework [50] and the Canadian Health Services Research Foundation (CHSRF) Self Assessment Tool [51]. While the participant in this study on whom an initial assessment was conducted was either a program manager or director responsible for making decisions related to healthy body weight promotion in children, we believe post-study it would have been more effective to have multiple senior decision makers complete this assessment and then have them discuss their perceptions in a facilitated, focus group session. The KB monitored participant status across all three levels and revisited plans of action with participants half way through and at the end of the one year intervention.

At the individual level, the KB noted the participant's position in the organization; length of time in the current position; perceived decision-making authority; values; preferences and attitudes towards the use of research evidence in decision making; informational needs; and knowledge and skills related to EIDM. Factors assessed at the organizational level included: perceived value the organization placed on research use (EIDM culture); existing infrastructure support for EIDM, such as financial, human, and other resources (*i.e*., access to computers, electronic databases, full text versions of systematic reviews and other evidence documents); incentives to promote EIDM; organizational decision making style; staff training in critical appraisal and research use; extent of recent restructuring and staff turnover; and quality improvement initiatives. Broader context or environmental factors assessed included: external networks; partnerships with researchers and other community stakeholders; and political priorities and influences. With respect to the evidence, the KB assessed common sources accessed by participants; their preferences for evidence sources and formats; as well as the type of decision made by participants and within which public health content areas.

#### Scanning the horizon

In order to facilitate participant access to the best available evidence, the KB was required to be knowledgeable of the most up-to-date evidence. Therefore, 'scanning the horizon' for new evidence and resources of interest to participants, as well as information related to KBs and brokering networks, was an important activity. This activity involved maintaining subscriptions to related list serves, electronic distribution lists, and e-table of contents alerts from relevant journals. The KB also subscribed to applications such as Really Simple Syndication (RSS) on specific journals and websites. RSS regularly checks for new content, downloading and sending any updates that it finds directly to the subscriber. This saved the KB a significant amount of time directly searching for new evidence.

#### Knowledge management

A good system for knowledge management was essential for effective and efficient knowledge brokering given the volume of information the KB exchanged with participants. By employing various technological applications and traditional filing systems, timely access to and retrieval of this large volume of information was facilitated. 'Must-have' technological applications included: client information management (contact and distribution lists, email filing, and journaling to aid in tracking client-related activities); reference management database software; and extensive bookmarking and categorization of relevant websites.

#### Knowledge translation and exchange

The majority of the KB's time was spent facilitating KTE. This was achieved by developing and maintaining a trusting relationship with participants, regular interaction with the research team and other key stakeholders; assisting with the writing and dissemination of tailored messages; and site visits to public health departments. The KB-initiated communication with participants occurred at a minimum, once per month, and more frequently as requested. One type of evidence transferred and translated by the KB in this study were the results of rigorous systematic reviews, available through the internet at health-evidence.ca, evaluating the effectiveness of interventions to promote healthy body weight in children. Also provided to them through health-evidence.ca were short summaries of each of the reviews that highlighted implications for public health policy and practice. The content and format of these summaries were developed based on extensive consultation with Canadian public health decision makers [35] and formed the content of the tailored messages sent to participants in both the tailored messages and KB intervention groups of the RCT. The KB was responsible for disseminating these summaries electronically as well as in hardcopy to participants in the KB group, along with other relevant evidence as needed or requested. The summaries were disseminated electronically as well as in hardcopy. The KB also sent the full text articles of the systematic reviews to those in the KB intervention group.

The KB also offered a site visit to each public health department in the KB intervention group. The purpose of the site visit was to facilitate the building of a trusting relationship between the health department and the KB, as well as to enable the KB to learn more about the local context. This enabled KB services to be tailored to the specific needs of each local environment. Furthermore, the activities conducted by the KB during each site visit then varied according to specific needs and goals identified by each health department. The number of public health professionals participating in the site visits ranged from one (the actual participant in the study) to entire healthy lifestyle or chronic disease prevention divisions of 25 to over 100 public health professionals. In many cases, the KB participated in team program planning sessions and assisted in the interpretation of evidence from the tailored messages and its incorporation into local program plans. The KB also conducted training sessions in many health departments to assist participants and their colleagues in developing their capacity to be critical consumers of information. In many instances, participants brought the KB to the communities served by their health department. It was during these visits that the KB learned more about the local realities and how these realities impacted on program planning and service provision.

#### Network development, maintenance, and facilitation

During baseline assessments, the KB identified the health promotion and obesity prevention networks with which participants were engaged. After the priorities, needs, and strengths for each participant and health department were identified, the KB informed participants of additional networks relevant and available to them. As well, the regional workshops provided opportunities for participants to connect with others from their region and webinars provided a virtual networking forum.

#### Facilitating knowledge and skill development

Opportunities to facilitate knowledge, skills development, and capacity for EIDM occurred during all interactions with the KB, at the individual (email, telephone, site visit) and group level (site visit, regional workshop, webinars). In many cases, participants sought the KB's advice on the methodological quality of an article, report, practice guideline, and/or program evaluation. The KB's role was to assist participants in critically appraising the quality of the evidence, and if the evidence was of high quality, to help identify implications for local programs and policies.

The three main goals of the regional workshops were to: present the results of the systematic reviews disseminated as part of the intervention in the RCT, facilitate discussion concerning the results, and identify implications for local program and policy development; provide participants with an opportunity to engage in individual and joint problem-solving related to EIDM; and provide face-to-face contact with the KB in order to promote KB credibility and to establish trust with participants.

Webinars provided opportunities for professional development, dialogue, networking, and knowledge exchange. During these sessions, participants discussed the steps of the EIDM process (identify an issue, identify high quality evidence, preferably synthesized evidence, assess methodological quality of evidence, identify implications for local policy and practice, implement evidence into practice, evaluate impact), organizational barriers and facilitators, innovative ideas to promote EIDM within their organizations, as well as the evidence reported in relevant systematic reviews and the implications in light of their local context.

The KB acted as a positive role model and mentor for participants by establishing effective working relationships with each participant, assisting them to connect high-quality evidence with local program planning goals, giving constructive feedback and evaluating their progress in EIDM.

#### Assisting participants in promoting organizational change to support EIDM

Organizational factors such as culture, decision making processes, leadership, and resources have been shown to be important to EIDM [52-64]. The KB provided support to participants as they worked to promote a culture in their organization conducive to EIDM. Key activities the KB engaged in were:

1. Promoting internal knowledge-sharing (*e.g*., suggesting the use of circulated table of contents alerts via team email distribution, the inclusion of discussions about specific systematic reviews at team and management meetings, and desktop links to relevant resources).

2. Assisting with the development of targeted resources (*e.g*., briefing notes for senior management and community partner bulletins).

3. Encouraging the inclusion of EIDM components in performance measures, and professional development activities.

4. Encouraging managers to act as role models (*e.g*., including the use of evidence in the decision making process by having managers require evidence to support recommendations and pose critical questions related to information and ideas brought forward from staff).

5. Encouraging collaboration with public health librarians or the libraries of academic institutions to assist in the development of efficient search strategies; placing links to key resources on desktops of staff.

6. Presenting to senior management and municipal or regional counsellors.

The extent to which the KB conducted these activities varied across health departments, depending on where the organization was with respect to EIDM; in all cases the KB worked to promote self-sufficiency in the individual participant and health department at whatever point they were in the EIDM process

## Discussion

KBs represent an emerging human resource in the health sector. However, the evidence regarding their effectiveness in promoting EIDM is lacking. While there are many commonalities across activities of those in formalized KB positions, no one job description comprehensively defines the role, and the required qualifications may differ significantly, depending on the target audience. Furthermore, there is some evidence linking KB attributes (*i.e*., personality characteristics) to impact, drawing into question the generalizability of interventions and outcomes to other settings or KBs [41,65,66]. Yet, knowledge brokering is considered to be adaptable to different contexts [31,47], and KBs have been shown to be instrumental in facilitating and improving communication and knowledge sharing between key stakeholders [32]. They are also associated with facilitating learning [17,67-69]; building capacity to locate, appraise, and translate evidence into the local context [17,38,47]; improving the quality of evidence used in decision making [41]; and increasing interpretation of research findings and implications for action [40].

### Lessons learned

In this section, lessons learned by the KB herself, as well as the research team in implementing the year-long KB intervention, will be highlighted. First is the importance of conducting an in-depth assessment of both the participant and the organization as early in the project as possible. Optimally, this assessment should be conducted face-to-face, although the telephone can be used when resources are limited. Early one-to-one contact was instrumental in facilitating the development of the KB/participant relationship, and in essence, set the stage for all activities to follow. For example, the one-third of participants in the RCT who had very early contact with the KB appeared to become more engaged in the EIDM process, and utilized the KB services to a greater extent than those who did not 'meet' the KB until later in the study. A further 30% either did not engage with the KB at all, or to a very limited extent. There did not appear to be any differences between those who engaged early with the KB and those who didn't on their level of capacity for EIDM. Not every participant responded to KB communication right away, meaning some did not meet the KB until two to three months following initiation of the intervention. The in-depth assessments also allowed for tailoring of the KB services over the full duration of the study by identifying at baseline the knowledge, skill, resource, support, and organizational change needs among the public health decision makers.

A second key lesson was the importance of putting in place a mechanism (*e.g*., network) to promote interaction and knowledge sharing among participants and with the KB. The KB recognized that public health decision makers across Canada were struggling with similar issues related to healthy body weight promotion in children, requiring similar knowledge and research evidence. Upon reflection, the KB believed that a facilitated network supported by electronic means such as teleconferencing, webinars, or groupware enhancements (*e.g*., discussion forum, shared workspaces) would optimize limited time and resources to more efficiently address participants' needs. Through a facilitated network, literature searches could more easily be shared with multiple participants; critical appraisal of the evidence could be done collaboratively online; and interpretation and implications of the research evidence could be discussed. A networking forum provided participants with the opportunity to share their experiences in using the evidence, the activities in which they were engaged, and their impact on local program planning and on changing organizational culture. Similar ideas are reported in the literature [70], particularly from a systematic review [46] that reports that social networks and formal networking approaches enhance EIDM efforts.

A third key lesson relates to time. It became apparent during the RCT that knowledge brokering is even more complex than we expected (*e.g*., it takes longer to develop collaborative, trusting relationships; much more capacity development was necessary than anticipated), and that the process of developing capacity for EIDM among public health decision makers and health departments takes considerable amounts of time. While the time it took any given participant and health department to move from one step of the EIDM process to the next varied, what became evident was each step took longer than we anticipated (*e.g*., we estimated capacity development would require two to three months of the intervention rather than six months). In hindsight, it is more likely that a multi-year KB intervention is needed to adequately impact on organizations' capacity for EIDM and would require a longer-term commitment of financial and human resources.

The final key lesson relates to the KB interaction and style. It is believed that a greater degree of face-to-face interaction between the KB and the participants would have been useful for developing the relationship, tailoring interventions, and promoting EIDM capacity. Effective strategies are required to facilitate partnership development and encourage individuals to work collaboratively with KBs. In addition, it is believed that several participants from each health department should have been involved in the KB intervention, thereby creating a critical mass in the organization with the skills and capacity for EIDM. Lastly, the KB must be cognisant of many factors that may affect success, such as political and organizational changes, issues of confidentiality, competing interests and priorities, and turf issues within and between organizations.

### To where from here?

While several important lessons were learned along the way in regard to the implementation of the KB role, a number of recommendations for future research were also identified. Most importantly, studies are needed to evaluate the effectiveness of KBs in different settings and among different health care professionals. In addition, research is needed to explore the optimal preparation and training of KBs, as well as the identification of the KB characteristics most closely associated with KB effectiveness. Finally, much work is needed to better understand which combination of KB activities are associated with optimal EIDM outcomes, and whether the combination changes in different settings and among different health care decision makers. Other important questions that need to be addressed include:

1. Is there an optimal dose for knowledge brokering?

2. What are effective strategies to promote participant engagement?

3. Is there a critical level of engagement between the organization and the KB that is associated with changing organizational culture?

4. Would KB facilitation of a network of public health decision makers improve the use of evidence in decision making, capacity development, and organizational change?

5. How important are KB attributes to the success of KB interventions?

## Conclusion

As the KB role developed during the RCT, central themes that emerged as particularly important included giving more attention to the time it takes to build trusting relationships and build skills and capacity for EIDM among public health decision makers, key attributes and responsibilities of KBs, and suggestions for improving the role in future activities. Finally, several suggestions for future research in this field were identified. The novelty of the KB role in public health provided a unique opportunity to assess the need for and reaction to the role and its associated activities, and clearer direction on how to move forward with the role have been identified.

## Competing interests

The authors declare that they have no competing interests.

## Authors' contributions

MD conceived of the study, participated in the analysis and drafted the manuscript. PR provided the intervention and assisted in draft of the manuscript. DC, SH, RC, LO, KD, SM, and SH consulted on the intervention as it was designed and provided, and participated in review of the manuscript. All authors read and approved the final manuscript.
